# 加速肺康复外科临床实践及证据

**DOI:** 10.3779/j.issn.1009-3419.2017.06.01

**Published:** 2017-06-20

**Authors:** 国卫 车

**Affiliations:** 610041 成都，四川大学华西医院胸外科 Department of Thoracic Surgery, West China Hospital, Sichuan University, Chengdu 610041, China

**Keywords:** 加速康复外科, 加速肺康复, 肺外科, Enhanced recovery after surgery, Enhanced lung recovery after surgery, Lung surgery

## Abstract

加速康复外科（enhanced recovery after surgery, ERAS）方案临床应用的多样性，取决于病种和团队而不同。肺术后都有哪些证据有利于加速肺康复（enhanced lung recovery after surgery, ELRAS）的临床实践呢？本文主要从围手术期可采用的措施及方法进行回顾性分析：术前强调教育及高危因素的评估及预防，术中微创手术及流程优化，术后关注以疼痛为主的症状管理。

## Abstract (en)

The clinic application pathway is a variant of the enhanced recovery after surgery (ERAS), which based disease and team. What are the published evidence is conducive to enhanced lung recovery after surgery (ELRAS) in clinical implementation. This article mainly from the perioperative management can adopt the measures and methods were retrospectively analyzed. The measures of education and assessment of the risk factors and prevention is emphasized in preoperative. The minimally invasive surgery and process optimization is adopted by surgeon. Postoperative focus on symptom management, such as pain is the first attention.

加速康复外科（enhanced recovery after surgery, ERAS）是医学理论和外科技术发展的必然结果，它不但关注减少对机体的应激反应，同时也重视对手术进行风险评估和干预，优化治疗共存病症包括心血管、呼吸系统和/或肾脏疾病，同时治疗、维持患者在围手术期重要器官功能，了解和处理患者存在的社会和行为因素，进而达到临床上降低并发症和缩短住院时间的目的^[1]^。目前，ERAS已在骨科、乳腺外科、心胸外科、胃肠外科、妇产科等多个外科领域开展且取得显著的临床结果^[2]^。国内外已发布多种术后ERAS指南或专家共识，如胃切除手术，肝胆胰手术等^[3]^。肺外科患者均存在术前伴随疾病多，术中麻醉、单肺和肺挫裂伤等使术后并发症发生率高，但近年微创技术和精准切除、损伤控制和流程优化的现代外科理念为ERAS的施行奠定了理论和实践基础^[4, 5]^。目前，多家医疗中心开始在肺手术患者中施行ERAS，并取得了一定临床效果。但是国内外均无一致的针对肺手术的ERAS方案来指导临床实践^[6]^。因此，有必要总结国内外该领域研究进展及专家经验，探讨加速肺康复（enhanced lung recovery after surgery, ELRAS）可能实现的途径，旨在为实现我国肺外科手术ERAS的规范化、标准化提供参考意见。

## 1 加速肺康复外科术前可采取的措施

### 1.1 术前宣传教育

术前通过集体或面对面交流，书面（宣传册）或多媒体方式，告知患者围术期各项相关事宜，包括^[7, 8]^：①术前戒烟或肺康复训练的意义及方法；②告知患者ERAS方案的目的和主要项目，鼓励患者术后早期进食、早期活动、宣传疼痛控制及呼吸理疗等相关知识，提高依从性；③告知患者麻醉和手术过程，减轻患者对麻醉和手术的恐惧和焦虑；④告知患者预设的出院标准；⑤告知患者随访时间安排和再入院途径。

### 1.2 术前肺功能评估

静态肺功能检测（pulmonary function test, PFT）不能正确评价患者的运动肺功能及运动耐力，且不能发现术前可能并存的高危因素。建议增加亚极量运动试验如爬楼试验，心肺运动试验或6分钟步行试验^[9, 10]^。

### 1.3 术前呼吸道准备

从目前已有研究来看，需要术前准备的人群有：①戒烟：至少戒烟2周以上，最好是4周^[11]^。并进行合适的呼吸道准备。②术前肺康复训练：对于高龄、合并中到重度慢性阻塞性肺疾病患者，吸烟史大于800年支患者，建议术前进行肺康复训练，如消炎、平喘等，雾化吸入糖皮质激素类或支气管扩张剂等；激励式肺量计吸气训练等^[6, 12-16]^。

### 1.4 术前禁食

传统围术期处理方案提倡术前禁食12 h、禁水6 h，认为可降低术后吸入性肺炎的发生率，但缺乏相应证据。同时有研究结果表明：禁食过夜可引起胰岛素抵抗和术后不适^[17]^。一项纳入了22项随机对照研究的*meta*分析结果表明：术前2 h进流质食物并未增加并发症发生率^[18]^。此外，术前避免长时间禁食可减轻术前不适。

### 1.5 术前心理疏导或镇静

术前心理疏导有助于降低术前焦虑，传统上术前晚应用镇静药物。但并无证据表明麻醉前使用抗焦虑药物能使术后疼痛减轻，反而使麻醉复苏困难或复苏后处于嗜睡状态^[19]^。因此，不主张在术前应用抗焦虑药物。

### 1.6 预防性抗菌药物使用^[20]^

有充分的研究证据支持术前预防性使用抗菌药物，认为其可降低手术部位感染发生率。主张切开皮肤前0.5 h-1.0 h或麻醉开始时给予抗菌药物，推荐静脉给药，且抗菌药物有效覆盖时间应包括整个手术过程。如手术时间 > 3 h或超过所用抗菌药物半衰期的2倍，或成年患者术中出血量 > 1, 500 mL，术中应追加单次剂量。抗菌药物可根据国家卫生和计划生育委员会指南选择，但预防性使用有别于治疗性使用。总体来说，预防性使用的抗菌药物应覆盖所有可能的病原菌。

## 2 加速肺康复外科术中需优化的流程

### 2.1 术中预防低体温

多项*meta*分析和临床随机对照研究结果均表明：避免术中低体温能降低切口感染、心脏并发症、出血和输血等发生率^[21]^。此外，术中低体温会影响药理及药代动力学，影响麻醉复苏。因此，术中应积极避免低体温发生，保持体温≥36℃。

### 2.2 目标导向性静脉补液

对于围术期患者，既应避免因低血容量导致的组织灌注不足和器官功能损害，也应注意容量负荷过多所致的组织水肿和心脏负荷增加。针对不同患者的个性化目标导向性补液治疗（goal directed fluid therapy, GDFT）可维持患者合适的循环容量和组织氧供，达到加快术后康复的目的^[22]^。有研究结果显示：GDFT的临床参考指标很多，实施过程中，需要连续、动态监测，维持血压下降幅度≤正常值的20%，心率加快幅度≤正常值的20%，中心静脉压（central venous pressure, CVP）为4 cm-12 cm H_2_O，尿量维持在 > 0.5 mL/(kg·h)，血乳酸≤2 mmol/L，中心静脉血氧饱和度（systemic central venous oxygen saturation, ScvO_2_） > 65%，每搏出量变异度≤13%。由于大部分患者术后可进食，故可以在术后尽早停止静脉补液。

### 2.3 术中入路和切口选择

手术入路和切口以能良好显露手术野为准，开放手术或胸腔镜手术都适用，微创手术首选。

### 2.4 术中尿管留置

术中留置尿管不但可引起患者不适，也易导致术后清醒时患者麻醉期苏醒期躁动和不良事件^[23]^，术后尿路感染和降低患者舒适度，并限制术后早期活动^[24, 25]^。麻醉后置尿管，而在患者完全清醒前拔掉尿管。若患者无尿道外伤或手术史、中重度前列腺增生或下腹部手术史，估计麻醉时间小于4 h，可以不置尿管^[25, 26]^。

### 2.5 胸腔引流管放置

胸腔引流管留置主要是防止术后胸腔积气、积液^[27]^。有研究^[28-30]^表明，单管（28 F、32 F、36 F）或细管（14 F、16 F、18 F）引流效果不劣于双管或粗管引流，且有助于患者术后活动、减少引流量、增加舒适度和引流管口愈合。故不强求常规不放置引流管，涉及胸膜腔闭锁、全肺切除及脓胸等手术仍推荐放置引流管，同时主张在无漏气、肺复张的情况下早期拔除引流管。

## 3 加速肺康复外科术后需要关注的症状

### 3.1 术后镇痛

80%的患者术后经历中重度疼痛，术后良好镇痛可缓解紧张和焦虑，且提高早期活动等依从性，降低静脉血栓和肺栓塞风险等。因此，术后镇痛是ERAS的重要环节，而“手术无痛”被视作ERAS的终极目标之一。预防镇痛，即在疼痛出现前采取镇痛措施以减缓术后疼痛的发生，其始于外科手术前，覆盖整个术中和术后，并按时有规律地给予镇痛药物。对于镇痛药物的选择，阿片类药物的不良反应较大，如影响肠功能恢复、呼吸抑制、恶心、呕吐等，应尽量减少使用^[31]^。近年来，联合应用阿片类与非阿片类药物使患者不良反应减少。非甾体类抗炎药物（non steroidal anti-inflammatory drugs, NSAIDs）被美国及欧洲多个国家的指南推荐为基础用药，建议若无禁忌证，首选NSAIDs，其针剂可与弱阿片类药物联合应用，片剂作为口服续贯镇痛药物^[32]^。在NSAIDs针剂的选择上，因非选择性NSAIDs可能增加出血风险和应激性溃疡发生率，推荐使用选择性环氧化酶-2（cyclooxygenase-2, COX-2）抑制剂、以降低出血风险。多模式镇痛采用硬膜外阻滞麻醉、患者自控镇痛泵（patient control analgesia, PCA）肋间神经阻滞等。术后采用多模式镇痛，以选择性COX2抑制剂，非选择性NSAIDs或对乙酰氨基酚为基础用药，包括PCA、NSAIDs针剂按时注射5 d-7 d和NSAIDs续贯镇痛等。具体措施：根据预防、按时、多模式镇痛理念，术前1 d-3 d使用NSAIDs，术后采用多模式镇痛，包括PCA、TAP阻滞、NSAIDs针剂按时注射5 d-7 d和NSAIDs续贯镇痛。

### 3.2 预防肺动脉栓塞^[33]^

肺外科术后肺动脉栓塞发生率约为1%，后果严重，死亡率高。预防性抗血栓形成措施包括基础预防、机械预防和药物预防。基础预防即早期活动；机械预防常用措施是间歇性空气加压（intermittent pneumatic compression, IPC）；药物预防有普通肝素、低分子肝素（low-molecular-weight heparin, LMWH）、阿司匹林等。LMWH与普通肝素比较，前者出血风险低，患者依从性高，可有效降低血栓形成风险，比IPC机械抗凝效果更佳。在排除出血风险的情况下，建议使用LMWH至术后可活动甚至直到出院为止；术前根据Caprini评分，选择相应预防性抗凝措施：Caprini评分≥4分，建议使用术前6 h-12 h应用1次LMWH直到出院。

### 3.3 预防恶心呕吐

术后恶心呕吐为常见麻醉不良反应。早期活动、进食及不应用或少用吗啡类或阿片类药物能减少术后恶心呕吐的发生。

### 3.4 术后饮食与营养

术后饮食建议以清淡或MCT为主，尤其是胃肠功能恢复以前。研究^[34]^发现MCT饮食不但有助于胃肠功能快速恢复，也可以减少胸腔引流量。

### 3.5 引流管拔除

早期拔除各种引流管，包括导尿管。有研究结果^[23, 26]^显示：长期留置导尿管增加尿路感染等风险。因此，建议术后即刻或24 h内拔除导尿管。

### 3.6 早期活动

早期活动指有目标地合理规划的活动。长期卧床会增加肺部感染、栓塞等并发症发生率。早期活动促进肌肉骨骼系统、呼吸系统等多系统功能恢复，可预防肺部感染、褥疮和深静脉血栓形成，同时促进胃肠功能恢复。早期活动目标的达成有赖于术前宣传教育、施行多模式镇痛和早期拔除引流管^[11]^。因此，进行合理规划的早期活动安全有益。推荐术后建立每日活动目标，逐日增加活动量。

### 3.7 术后肺康复训练

若术前合并高危因素，建议术后仍继续进行康复训练，方案同术前^[11]^。

## 4 结语

ERAS方案的目的主要是加速患者术后安全康复，并非仅追求术后住院时间的缩短。因此、围术期处理措施的施行必须在循证医学或真实世界数据或证据指导下进行，以使患者受益为目的。ERAS方案的重点在于经过合理的处理措施，患者并发症发生率降低，在此基础上术后住院时间才能安全缩短。不同地区、不同疾病、不同手术、不同患者ERAS的具体方案并不能一概而论。
